# External Validation of a Clinical Prediction Tool for the Use of Manual Therapy in Patients With Temporomandibular Disorders

**DOI:** 10.1111/joor.70092

**Published:** 2025-11-11

**Authors:** G. Asquini, V. Devecchi, G. Borromeo, P. Tessera, A. Russillo, A. Michelotti, R. Bucci, D. Falla

**Affiliations:** ^1^ Centre of Precision Rehabilitation for Spinal Pain (CPR Spine), School of Sport, Exercise and Rehabilitation Sciences, College of Life and Environmental Sciences University of Birmingham Birmingham UK; ^2^ Italian Stomatologic Institute Craniomandibular Physiotherapy Service Milan Italy; ^3^ Dental and Maxillo‐Facial Surgery Unit, IRCCS Ca' Granda Ospedale Maggiore Policlinico di Milano Milan Italy; ^4^ Section of Orthodontics, Department of Neurosciences, Reproductive Sciences and Oral Sciences University of Naples ‘Federico II’ Napoli Italy

**Keywords:** manual therapy, pain, precision medicine, prediction, temporomandibular disorders

## Abstract

**Background:**

Manual therapy (MT) is frequently used to manage temporomandibular disorders (TMDs), yet patient responses vary significantly. Predictive models may help clinicians tailor treatments to individual patients.

**Objectives:**

The primary aim of this study was to externally validate a previously developed prediction model for identifying patients with TMDs who are more likely to benefit from MT. Additionally, new prognostic models to predict outcomes at a one‐month follow‐up were developed.

**Methods:**

A cohort of 124 adults with a diagnosis of a TMD received a four‐week MT program (one session per week) applied to craniomandibular structures. Predictors collected at baseline included clinical and psychosocial variables: pain during mouth opening, pain localisation, treatment expectations, and the central sensitisation inventory (CSI). The primary outcome was a ≥ 30% pain reduction post‐treatment. Model performance was assessed using discrimination, calibration, and decision curve analysis. New models predicting one‐month outcomes were developed and internally validated via bootstrapping.

**Results:**

The original model showed strong discrimination and overall fit in the validation cohort (AUC = 0.95, *R* ^2^ = 0.75). Except for pain location, the predictors in the original model also showed excellent discrimination in the developed model based on the outcome at the one‐month follow‐up (AUC = 0.96). A significant interaction between treatment expectations and CSI was found, with high CSI negatively affecting the outcome in people with positive treatment expectations.

**Conclusions:**

This study externally validated a clinical prediction model for pain reduction in people with TMDs following MT, confirming previously identified predictors of good outcome such as pain during mouth opening, positive treatment expectations, localisation of pain in the craniocervical region, and lower CSI scores. A link to a web‐based calculator of the prediction model is provided. The interaction between treatment expectations and CSI suggests that CSI plays a key role in shaping treatment response, modulating the influence of treatment expectation. Future studies with a control group are needed to confirm these results and distinguish true treatment effect modifiers from general prognostic factors.

**Trial Registration:**

ClinicalTrials.gov ID: NCT03990662

## Graphical abstract

Predicting the response to manual therapy for people with temporomandibular disorders.

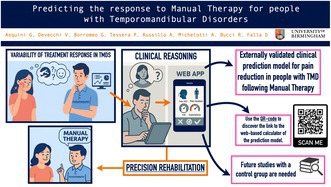

## 1 Introduction

Temporomandibular disorders (TMDs) are musculoskeletal disorders affecting approximately 10% of adults in developed countries, with an increasing prevalence over the last 10 years [1, 2, 3]. Patients suffering from TMDs often experience orofacial pain, altered jaw movements, and potential joint sounds. These signs and symptoms often occur along with neck and upper back pain, which can further impact quality of life [4, 5, 6, 7]. As described in the OPPERA study, risk factors for TMDs are varied and intricate, involving physical, psychological, and social factors [8, 9, 10].

Current clinical guidelines recommend an integrated biopsychosocial approach for the management of people with TMDs in order to address such complexity [11, 12]. Effective management typically involves multidisciplinary and conservative therapies, including physical therapy techniques such as manual therapy (MT) [11, 13]. These approaches are reported to be effective for reducing pain and improving jaw function [13, 14, 15, 16, 17]. MT, particularly when applied directly to temporomandibular structures, has shown positive outcomes in reducing pain and enhancing jaw movement [13]. Nevertheless, despite its general effectiveness, patient responses to MT can vary considerably [13, 14, 15, 16, 17].

When treating people with TMDs, the selection of an appropriate intervention depends on diverse clinical factors and individual attitudes towards different treatments [18]. One way to optimise treatment outcomes is by identifying patients more likely to benefit from specific therapeutic approaches. According to Hancock et al. [19], treatment effect modifiers are variables that help distinguish subgroups of patients responding differently to a given intervention [19]. The development of predictive tools for different musculoskeletal disorders has been shown to improve clinical decisions and allow treatments to be better tailored to individual patient needs [20, 21, 22]. Additionally, innovative machine‐learning‐based studies showed the utility of predictive tools to enhance clinical decision‐making for patients with TMDs [23]. Developing treatment‐related predictive algorithms for patients with TMD could potentially improve treatment personalisation, potentially addressing the variability in MT response for such patients.

Previously, we conducted a prospective observational study to develop a clinical prediction model which identifies patients who would likely benefit from MT [22, 24]. A pain intensity reduction equal to or greater than 30% after a programme of MT was defined as a good outcome [22, 24]. This model identified four key predictive factors which were pain intensity during mouth opening, positive expectations towards treatment outcomes, pain primarily localised in the craniocervical region, and lower scores on the Central Sensitisation Inventory (CSI). Additionally, patients with shorter pain duration and greater limitations in mouth opening at baseline were more likely to experience functional improvements after a MT intervention [22, 24]. However, before applying this prediction model in routine clinical practice, external validation is essential. External validation tests the model in a new, independent group of patients to confirm its accuracy, reliability, and usefulness [25]. This step is crucial for establishing confidence in the prediction model and ensuring its generalisability across different patient populations and clinical environments [26].

The current study aims to externally validate our previously developed clinical prediction model [22, 24]. We tested the model's predictive capability in a new, separate group of patients diagnosed with TMDs [22, 24]. Consistent with the development study, the primary outcome was the change in pain intensity from baseline, with a ≥ 30% reduction indicating a good outcome [22, 24]. Additionally, the secondary outcome was patient‐reported function measured with the Patient‐Specific Functional Scale. Validating this model would provide clinicians with a robust, evidence‐based tool to predict patient outcomes in response to MT, ultimately facilitating more personalised treatment strategies and leading to enhanced patient care. Additionally, we aimed to develop new prediction models to assess whether the clinical predictors identified in the original study demonstrate good performance in predicting outcomes of interest at a 1‐month follow‐up after completion of the MT intervention.

## 2 Methods

### 2.1 Source of Data

This prospective observational study recruited participants from the temporomandibular joint (TMJ) unit of the Italian Stomatologic Institute (Milan, Italy), the same setting as the original study. Eligible individuals with a diagnosis of a TMD, based on the Diagnostic Criteria for TMDs (DC/TMD), were included [27]. The study followed established methodological standards for prediction model development and validation, as outlined in the Transparent Reporting of a multivariable prediction model for Individual Prognosis or Diagnosis (TRIPOD) statement (the TRIPOD checklist is reported in File S1) [28]. Ethical approval was obtained from the Ethics Committee of the Fondazione IRCCS Ca' Granda Ospedale Maggiore Policlinico (approval no. “534_2019bis”). The study was conducted in compliance with the Declaration of Helsinki. Baseline data were collected prior to the start of the first MT session. Participants completed a four‐week MT intervention, consisting of one session per week as performed in the original study. Outcome measures were assessed at baseline, at the end of the fourth MT session (post‐treatment) and one month later (1‐month follow‐up) [22, 24].

### 2.2 Setting and Participants

Participants were enrolled at the Italian Stomatologic Institute, a dental hospital in Milan (Italy) from April 2023 until December 2024. All patients referred to the TMJ Unit who met the eligibility criteria were considered for inclusion. The same setting and eligibility criteria used in the model development study were applied in this validation study [22, 24].

#### 2.2.1 Eligibility Criteria

Inclusion criteria were: (1) age ≥ 18 years; (2) diagnosis of a TMD based on the DC/TMD criteria [27]; (3) no treatment received for their TMD within the previous 6 months [22, 24]; (4) sufficient ability to understand and communicate in Italian; (5) capacity to provide written informed consent.

Exclusion criteria included: (1) TMD‐related pain associated with rheumatoid or inflammatory arthritis; (2) physical (e.g., facial paralysis, neurological conditions, neuropathic pain) or mental health issues (e.g., cognitive impairment, psychiatric disorders) likely to affect outcome assessments; (3) initiation of another TMD treatment during the study period (e.g., pharmacological, oral appliances); (4) use of medications influencing neuromuscular function for conditions other than a TMD; (5) malignancy; (6) pregnancy; (7) substance or alcohol abuse.

Both the development and the external validation were conducted within the same clinical setting, involving clinicians with similar training and experience. While this ensures methodological consistency and tests the reproducibility of the prediction model, it limits transportability to other clinical settings and levels of expertise [29]. The implications for generalisability are addressed in the Discussion.

### 2.3 Recruitment

**FIGURE 1:**
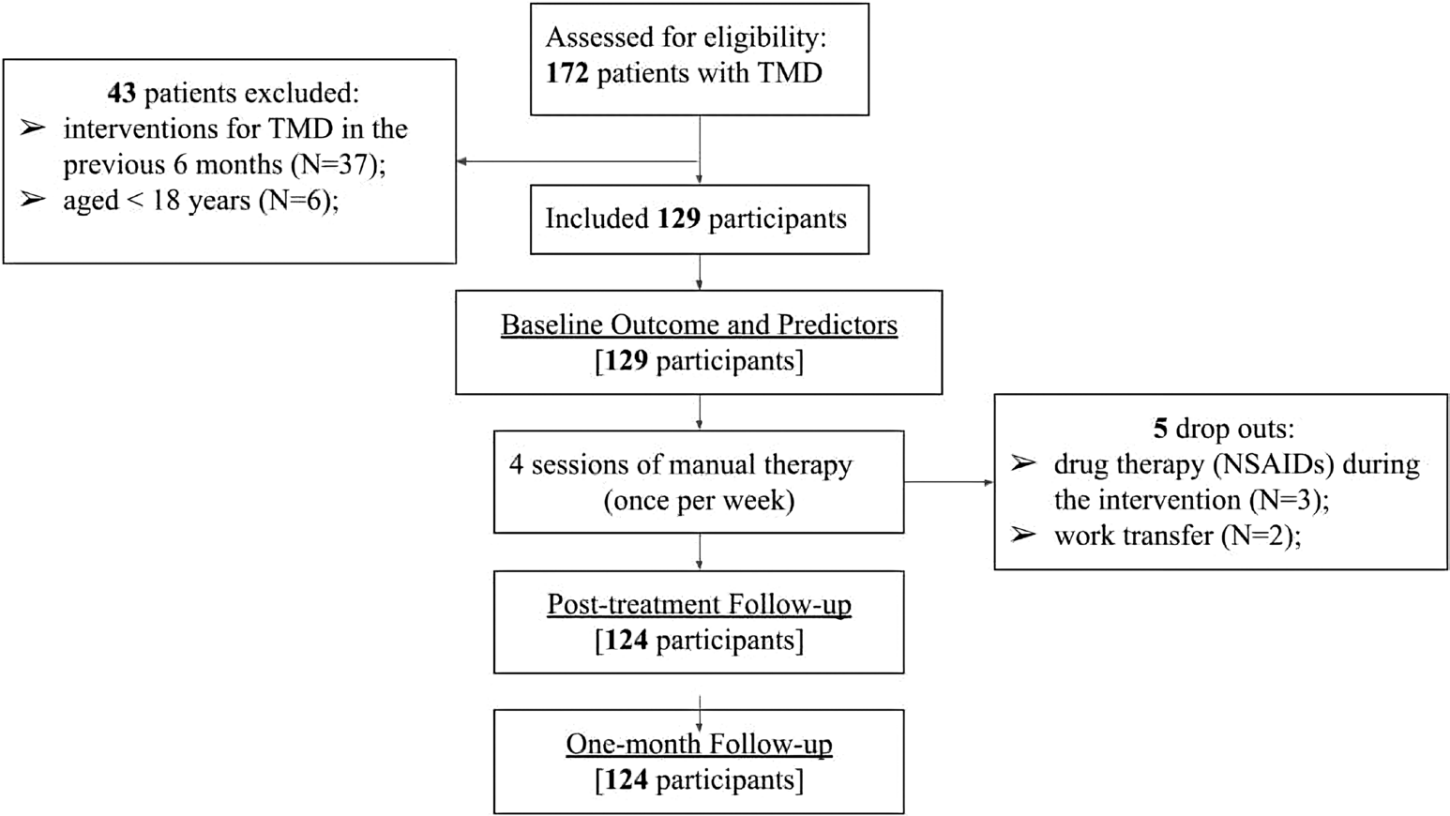
The flow of participants through each phase of the study.

All patients referred for a possible TMD to the TMJ Unit at the Italian Stomatologic Institute were screened for eligibility. In total, we screened 172 patients with TMDs, with 129 participants meeting the eligibility criteria. Diagnosis was confirmed using the Italian version of the DC/TMD protocol, administered by two experienced dentists with over 10 years of clinical expertise in managing people with TMDs [30]. Participants who met the eligibility criteria received detailed information about the study procedures and provided written informed consent. Baseline assessments were then conducted by an independent physiotherapist with more than 5 years of experience in TMD management. The manual therapy sessions began within the same week. Outcome data were collected at three time points: before the start of treatment (baseline), after the final MT session (at 4 weeks), and again at a one‐month follow‐up (8 weeks from baseline). All evaluations were performed by the same independent physiotherapist assessor to ensure consistency, as in the previous study. All evaluations were performed by the same independent physiotherapist who did not deliver the MT intervention, reducing performance and treatment‐allegiance bias; however, the assessor was not blinded to baseline predictor data and collected both predictors and primary/secondary outcomes at all time points, which may have introduced detection/observer‐expectancy bias. The flow of participants through each phase of the study is summarised in Figure 1.

### 2.4 Intervention

The intervention was the same used in the original study and consisted of a programme of MT delivered over four consecutive weeks, with one session per week, delivered by the same physiotherapist. Each session involved hands‐on techniques targeting exclusively the temporomandibular structures. This treatment protocol had demonstrated effectiveness in the original development study and has been supported by similar approaches described in existing literature [31, 32, 33]. Based on the clinical examination, current evidence, and their own clinical reasoning, the treating physiotherapist selected the most appropriate MT techniques for each patient. Decisions were guided primarily by pain severity and irritability, the suspected pain sources and contributing factors identified during assessment. The intervention could include TMJ mobilisations with directional glides (e.g., inferior/caudal, anterior/ventral, anteroposterior, and lateral/medial translations) and soft‐tissue techniques targeting the masticatory muscles (e.g., trigger‐point release or myofascial techniques). The overarching aims were to decrease pain and muscle hypertonicity, restore joint mobility, and improve jaw function [22, 24, 34]. The intervention was part of the standard clinical care provided at the TMJ Unit of the Italian Stomatologic Institute. All MT sessions were executed by two physiotherapists with over 5 years of clinical experience in MT for TMDs. These physiotherapists were not involved in participant recruitment or outcome assessments. No treatment was delivered to adjacent areas such as the cervical spine. During the sessions, physiotherapists addressed participants' questions and offered general advice as needed. Participants were withdrawn from the study if they initiated other treatments for their TMD (e.g., oral appliances, drugs) or sought care for acute symptoms in other regions (e.g., cervical pain) during the intervention period. Adherence was defined as the number of MT sessions attended out of four (4/4, 3/4, 2/4, 1/4), recorded in therapist logs at each visit.

### 2.5 Outcome Measures

The outcome of interest was pain intensity, as patients with TMD frequently report pain as their most disabling symptom [4]. Pain intensity was evaluated using the Visual Analogue Scale (VAS), calculated as the mean of current pain, average pain over the past week, and worst pain in the past week [22, 24]. This scale has shown good reliability and validity in clinical trials focused on pain assessment [35, 36]. As in our previous study and following recommendations from the Initiative on Methods, Measurement, and Pain Assessment in Clinical Trials (IMMPACT), a reduction of 30% or more in VAS pain intensity scores was considered clinically significant, and this was considered the primary outcome for the prediction model [22, 24, 37]. Changes in pain intensity and changes in function were the secondary outcomes. Participants' function was assessed using the Patient‐Specific Functional Scale (PSFS) [38, 39], which is a self‐reported tool that has shown strong validity and reliability in populations with musculoskeletal disorders [40, 41, 42, 43, 44]. All outcome assessments were carried out by the same independent physiotherapist to minimise detection bias [45].

### 2.6 Predictors

The original prediction model identified four variables associated with pain reduction and two predictors of functional improvement in patients with TMD [22]. In the present study, the same independent physiotherapist who performed the outcome assessments also collected demographic and predictive variables at baseline, following the standardised procedures established in all previous studies [22, 24, 46].

#### 2.6.1 Pain During Maximal Mouth Opening

Pain intensity during maximal mouth opening was recorded using an 11‐point Numeric Rating Scale (NRS) [47]. Participants were asked: “Rate your pain by indicating the number that best describes pain during maximal mouth opening, with 0 meaning ‘No pain’ and 10 meaning ‘Pain as bad as you can imagine’” [37, 47]. The NRS has demonstrated strong reliability and validity in clinical research focused on pain [38].

#### 2.6.2 Central Sensitisation

Central sensitisation was evaluated using the Italian version of the CSI [48]. Only Part A was used, and it consists of 25 items assessing the frequency of various somatic symptoms on a scale from 0 (never) to 4 (always), with a total score ranging from 0 to 100 [49]. A score greater than 40 is considered indicative of central sensitisation [49]. The CSI has shown good test–retest reliability and internal consistency in both pain and non‐pain populations [46]. The Italian version has a Cronbach's alpha of 0.87, indicating acceptable internal consistency [48].

#### 2.6.3 Treatment Expectations

Expectations regarding treatment were assessed using the same procedure applied in the original study [22, 24]. Participants were asked to rate their level of agreement with the statement: “I believe that manual techniques applied to my jaw will significantly help to improve my pain.” Responses ranged from “Completely disagree” to “Completely agree.” Only responses of “Somewhat agree” or “Completely agree” were classified as positive expectations.

#### 2.6.4 Duration of TMD Pain

The duration of TMD‐related pain was documented in days, based on a combination of patient interviews and electronic medical records.

#### 2.6.5 Number of Pain Locations

Participants completed a pain drawing on standardised body charts depicting anterior and posterior views. Each anatomically distinct region that was reported as painful (e.g., jaw, head, neck, back, pelvis) was counted as one pain site. Details regarding chart design and scoring have been published previously [22, 24].

#### 2.6.6 Maximal Mouth Opening

Maximal mouth opening (MMO) was measured in millimetres using a ruler, with the patient in a neutral craniocervical position (sitting or supine), as recommended by the DC/TMD protocol [27, 30]. This measurement approach has demonstrated good intra‐ and inter‐rater reliability and was consistent with procedures used in the prior development study [22, 24, 27, 30, 50].

All predictor variables, along with demographic characteristics (age and gender), were collected at baseline. Pain during MMO and MMO were the only variables assessed at all time points.

### 2.7 Sample Size

Based on the proportion of events and non‐events in the original study, we aimed to recruit a minimum of 120 participants having at least 10 events per predictor variable. Since we recognised that we could not fully satisfy current guidance for external validation that recommends at least 100 events and non‐events to allow precise estimation of discrimination and calibration, we reported all performance metrics (c‐statistic, calibration‐in‐the‐large, calibration slope) together with 95% confidence intervals (95% CIs) to provide the associated uncertainty.

### 2.8 Data Analysis

#### 2.8.1 Data Handling

Predictor variables were operationalised and coded as defined in the original model development study and protocol, with pain intensity during MMO, dichotomised a priori to distinguish patients with no or minimal pain (VAS ≤ 2) from those with moderate to severe pain (VAS > 2), a common threshold used to identify pain that becomes functionally limiting and requires intervention. Similarly, pain extent was dichotomised based on the number of reported pain locations, categorising patients as experiencing localised pain (≤ 2 locations, including temporomandibular and cervical regions) or widespread pain (> 2 locations). These cut‐offs were chosen to enhance model interpretability and to limit the risk of overfitting that can arise from data‐driven categorisation. The dataset for this external validation study had no missing values for the predictors, thereby removing the need for data imputation. All statistical analyses were performed using R software. A *p*‐value ≤ 0.05 was considered statistically significant unless otherwise stated.

To evaluate the effectiveness of the programme of MT, the distribution of pain intensity data was evaluated and a paired *t*‐test or Wilcoxon‐signed rank test was used to compare the level of pain at baseline with the one reported after the intervention (post‐treatment) and one month later (1‐month follow‐up).

#### 2.8.2 External Validation of Post‐Treatment Models

To evaluate the relatedness of the external validation sample to the original development sample, we followed the methodology described by Debray et al. [51]. A logistic regression model was fitted with dataset membership (development vs. validation cohort) as the binary dependent variable. Independent variables included the full set of model predictors, baseline demographic characteristics (e.g., age, sex), and primary study outcome. A low discriminative ability of this logistic model for dataset membership, indicated by a c‐statistic approaching 0.50, would suggest high similarity between the samples and support an evaluation of model reproducibility. Conversely, a higher c‐statistic would indicate differences warranting careful consideration of transportability.

Previously developed prognostic models for outcomes assessed at the end of the MT intervention were externally validated in the current cohort. For the primary binary outcome of pain improvement (poor versus good), the linear predictor (*lp*) was first calculated for each participant in the validation cohort using the intercept and regression coefficients published for the original model. The probability of a good outcome was then calculated as *p*(*y* = 1) = 1/(1 + *e* ^−*lp*^). The distribution of *lp* in good and poor outcome events was examined and presented as recommended [29]. Overall model fit was evaluated using Nagelkerke's *R* ^2^ and the Brier Score. Discriminative ability was quantified using the c‐statistic (Area Under the Receiver Operating Characteristic Curve, AUROC) with 95% CIs. Calibration was assessed by examining calibration‐in‐the‐large (mean observed vs. mean predicted probabilities), the calibration slope, and visually with calibration plots comparing observed frequencies to predicted probabilities. Excellent calibration requires a calibration‐in‐the‐large close to 0 and a slope of 1. Finally, clinical utility was determined using decision curve analysis (DCA) to plot the net benefit against a range of threshold probabilities for a good outcome, comparing the model's utility against treat‐all and treat‐none strategies. Given that MT is a relatively low‐risk intervention, the primary range of clinically relevant thresholds was considered to be between 10% and 40% [37]. Indeed, the “harm” of treating a patient who will not benefit (a false positive) is low, consisting mainly of the financial cost and time for treatment, with negligible risk of serious adverse events. In contrast, the harm of not treating a patient who would have benefited (a false negative) is the continuation of significant pain and functional limitation. Given this favourable risk–benefit profile, clinicians and patients are likely to consider treatment worthwhile even at modest probabilities of success.

External validation was also conducted for models predicting continuous secondary outcomes, including the percentage of pain change and the change in physical function (disability points). For these models, overall fit was primarily assessed by the coefficient of determination (*R* ^2^) and the Root Mean Square Error (RMSE). Calibration for these continuous outcomes was assessed by regressing observed values on predicted values, examining the intercept (ideal 0) and slope (ideal 1) of this regression line, and visually inspecting observed versus predicted values using scatter plots.

Following the initial external validation performance assessment, model updating strategies were considered for all post‐treatment models if miscalibration or poor performance were identified. Potential updates included adjustment of the model intercept (recalibration‐in‐the‐large) or a more comprehensive recalibration involving adjustment of both the intercept and the global calibration slope.

The three prediction models to be validated are reported in Table 1, along with the coefficients for each predictor and performance obtained in the original study.

**TABLE 1:** Prediction model to externally validate in the new cohort.

Model name	Model calculation	Performance values after optimism adjustment
Responders model	1.40 − 0.08 * CSI − 0.31 * NPL + 1.63 * TE + 2.34 * PMO	Nagelkerke's *R* ^2^ = 0.58 c‐statistic = 0.89
Pain model	45.5 − 0.59 * CSI − 5.00 * NPL + 18.96 * TE + 20.43 * PMO	*R* ^2^ = 0.40 RMSE = 24.40
Function model	4.11 − 0.06 * MMO − 0.39 * LN (history tmd)	*R* ^2^ = 0.26 RMSE = 1.46

Abbreviations: CSI, central sensitisation inventory; MMO, maximal mouth opening; NPL, number of pain locations; PMO, pain during mouth opening; RMSE, root mean square error; TE, treatment expectation.

#### 2.8.3 Development of Prognostic Models for One‐Month Follow‐Up Outcomes

Prognostic models were developed for the primary (binary) and secondary (continuous) outcomes assessed at the one‐month follow‐up. The initial set of candidate predictors included those in the externally validated post‐treatment models. Linearity assumptions for continuous predictors were systematically evaluated using graphical methods (e.g., plotting residuals against continuous variables) and by testing for non‐linear transformations (e.g., restricted cubic splines with varying knots). Potential interaction terms between predictors were also considered. A backward elimination strategy, based on a change in the Akaike Information Criterion (AIC) equivalent to a *p*‐value of 0.157 for variable removal, was employed for predictor selection to balance model fit and parsimony.

Internal validation of developed models was performed using 1000 bootstrap replicates to estimate optimism‐corrected performance measures. For the binary follow‐up outcome model, these optimism‐corrected measures included the c‐statistic (with 95% CI), Nagelkerke's *R* ^2^, and the Brier Score. Calibration was also assessed by examining calibration plots. Similarly, for the continuous follow‐up outcome models, optimism‐corrected performance was characterised by *R* ^2^, RMSE, and visually assessing the regressed observed values on predicted ones.

## 3 Results

Five participants did not complete the intervention and dropped out from the study, and they were excluded from the analysis. Two participants dropped out due to work‐related travel, while the other three discontinued the study after initiating pharmacological treatment during the intervention. There were no differences in demographic variables or predictors between participants included in the final analysis and participants who dropped out (File S2). No post‐treatment outcome data were available for these five participants, so a complete‐case analysis was performed with the remaining 124 participants. Among completers, all 124 participants attended the full course of four sessions (4/4) within one month.

### 3.1 External Validation of the Original Prediction Models

A comparison between the sample of the original study and the validation cohort was conducted considering predictors, key demographic and primary outcome. The values for all variables in the two samples are reported in Table 2. After logistic regression with dataset membership as the dependent variable, with a c‐statistic of 0.68, the two cohorts were moderately different; therefore, some performance loss in the validation cohort was anticipated. The variables showing the larger difference between samples were age and score on the CSI, with a slightly older population and higher CSI scores in the validation cohort compared to the sample in the original study. Given the use of the same setting and the relatedness between the two cohorts, the study primarily tested reproducibility rather than transportability of the prediction model.

**TABLE 2:** Predictor variables and characteristics of participants in the validation cohort and the development study.

Variable	Statistic	Validation cohort (*n* = 124)	Development study (*n* = 90)
Age	Median [IQR]	38.00 [21.00]	34.00 [20.75]
Maximum mouth opening	Median [IQR]	31.00 [14.25]	31.00 [13.50]
Central sensitisation inventory	Median [IQR]	36.00 [18.25]	33.00 [19.75]
Pain duration	Median [IQR]	180.00 [300.00]	120.00 [295.00]
Pain baseline [0–100]	Mean (SD)	50.41 (15.44)	53.94 (18.84)
Gender	*N* (%) (female)	94 (75.80%)	74 (82.20%)
	*N* (%) (male)	30 (24.20%)	16 (17.80%)
Treatment expectation	*N* (%) (negative)	33 (26.60%)	17 (18.90%)
	*N* (%) (positive)	91 (73.40%)	73 (81.10%)
Number of pain locations	*N* (%) (≤ 2)	80 (64.50%)	57 (63.30%)
	*N* (%) (> 2)	44 (35.50%)	33 (36.70%)
Pain during mouth opening	*N* (%) (≤ 2)	37 (29.80%)	28 (31.10%)
	*N* (%) (> 2)	87 (70.20%)	62 (68.90%)
Outcome	*N* (%) (poor)	43 (34.70%)	27 (30.00%)
	*N* (%) (good)	81 (65.30%)	63 (70.00%)

**FIGURE 2:**
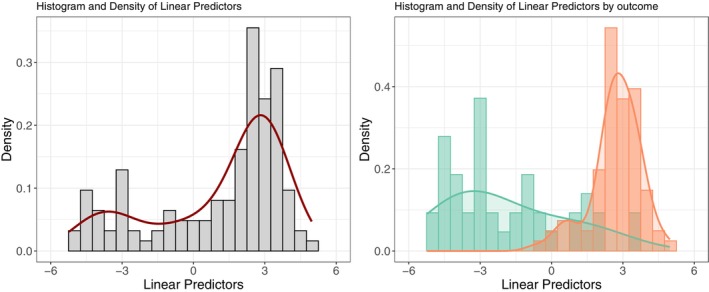
Distribution of the linear predictors for the outcome at the end of manual therapy programme. The colour indicates the linear predictors for people with good (red) and poor (green) outcome.

At the end of the MT intervention, 81 patients had a good outcome (i.e., percentage reduction in pain ≥ 30%) and 43 had a poor outcome, values below the recommended guidance of at least 100 events and non‐events, anticipating large CI in the model performance metrics. Assessed on a 0–100 VAS scale, the average reduction in pain was 24.2 (95% CI from 20.9 to 27.6, *p* < 0.001) which corresponded on average to a percentage of improvement of 49.8% compared with baseline. Of the 81 patients with a good outcome, 20 reported full recovery (i.e., 100% pain reduction). The prediction of patients having good versus poor outcomes after the MT intervention was conducted using the prediction model of the original study (Table 1). Linear predictors were computed for each participant and their distribution in the cohort and by outcome are presented in Figure 2, showing a bimodal distribution with the two peaks corresponding to the majority of people having good and poor outcomes. The relationship of pain during MMO and the number of pain locations, when treated on their original scales, with the primary outcome is presented as Files S3 and S4. These analyses show that the cut‐offs used for dichotomizing the variables do not lead to a loss of information and correspond to the transition between good and poor outcomes. Furthermore, model performance remained strong also after performing best/worst‐case analysis for missing data (File S5).

**FIGURE 3:**
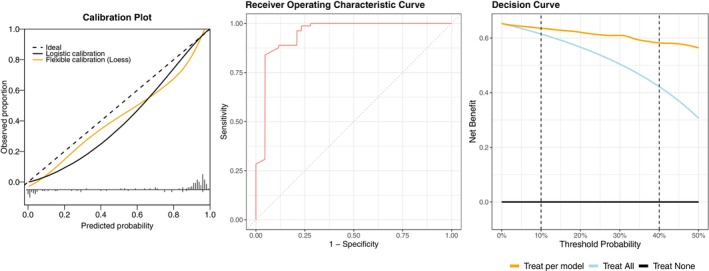
Calibration plot, discrimination curve, and decision curve of the responder model after external validation.

After comparing the predicted probability of the original model in the validation cohort with the observed outcome, the model showed high discrimination and overall fit with acceptable calibration. Specifically, the c‐statistic of the model was 0.95, 95% CI (0.90–0.99), the Nagelkerke's *R* ^2^ was 0.75 and a Brier Score of 0.076. Calibration is visually presented in Figure 3 along with the discrimination AUC‐ROC curve. Calibration‐in‐the‐large was −0.35 95% CI (−0.94 to 0.25) and the calibration slope was 1.21 with 95% CI (0.77–1.65). Finally, the decision curve analysis showing the higher net benefit of the validated model at different threshold probabilities compared with the “treat all” approach, is presented in Figure 3. Due to the small sample size risking resulting in a large error estimate and the acceptable calibration including 0 and 1 in the confidence interval of calibration‐in‐the‐large and calibration slope, respectively, the model was not updated.

**FIGURE 4:**
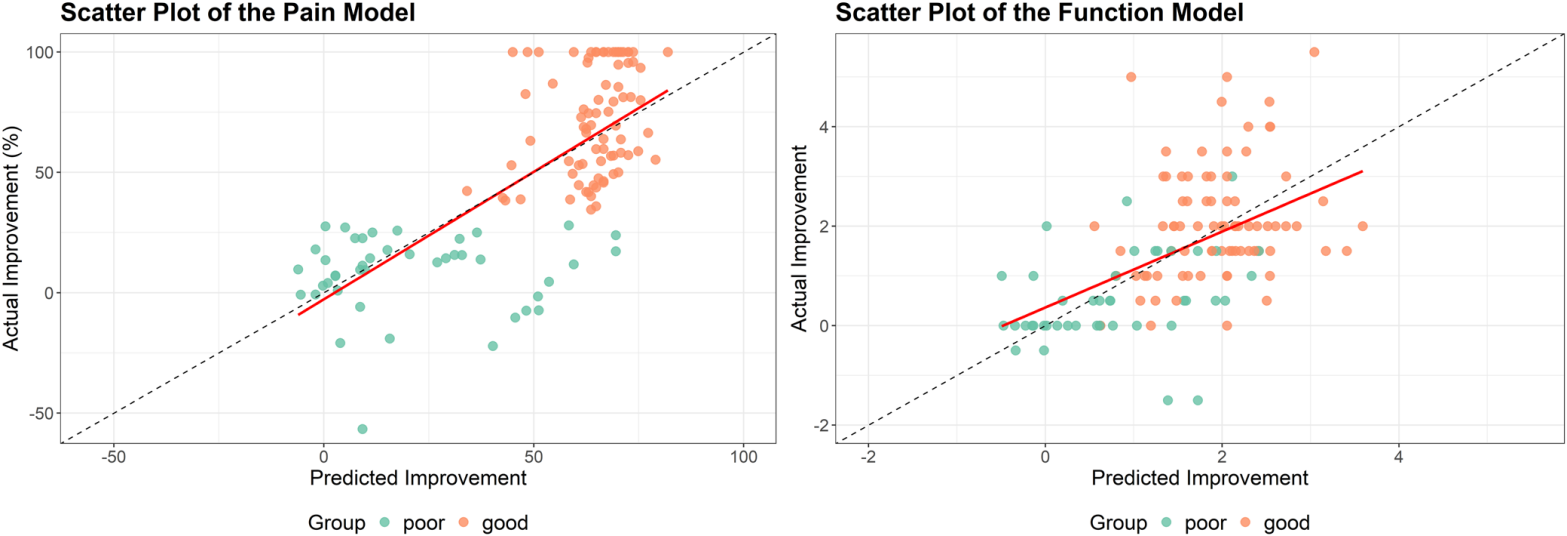
Overall fit of pain model and function model after external validation. Predicted versus observed values are shown. The function model is presented after update of the intercept.

The model predicting pain improvement after MT showed good overall fit as illustrated in Figure 4 which shows the predicted improvement against the observed improvement in the validation cohort. The regression line of prediction versus observed outcome showed high consistency with the ideal pattern, and it had −2.74 intercept and 1.06 slope on a percentage of change scale. The adjusted *R* ^2^ was 0.52 with a RMSE of 25.4.

The model predicting functional improvement after MT showed a moderate overall fit with an *R* ^2^ of 0.27 and a RMSE of 1.46. The graph of predicted improvement against the observed improvement in the validation cohort showed miscalibration with a low intercept of −0.34 and slope of 0.76. After recalibration of the model intercept, the RMSE was reduced to 1.13 and the new model prediction versus observed values are presented in Figure 4. Recalibration of other model parameters lead to minimal reduction in the RMSE, so the model with only intercept update was retained.

### 3.2 Development of New Prediction Models With Outcomes at the One‐Month Follow‐Up

**FIGURE 5:**
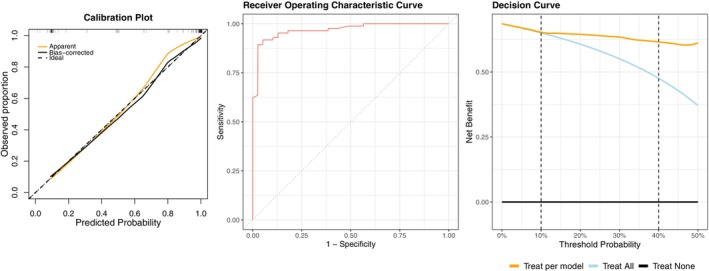
Calibration plot, discrimination curve, and decision curve of the responder model developed with outcome at one month follow‐up as dependent variable.

At the one‐month follow‐up, of the 124 patients, 85 reported a good outcome and 39 a poor outcome. The average reduction in pain was 29.9 (95% CI from 26.0 to 33.9, *p* < 0.001) which corresponded on average to a percentage of improvement of 61.1% with respect to baseline. Of the 85 patients with good outcome, 47 reported a full recovery (i.e., 100% pain reduction). To develop the prediction model, the same predictors of the validation model were considered. Due to the strong effect of the interaction between CSI and treatment expectations of the outcome, this was introduced in the initial model. After backward selection, the predictors retained in the final model were CSI, treatment expectations, pain during MMO and the interaction term between CSI and treatment expectations. Performance of the model was characterised by high discrimination and overall fit. After optimism adjustment, the c‐statistic was 0.96, Nagelkerke's *R* ^2^ was 0.76, Brier Score was 0.07, calibration‐in‐the‐large −0.10 and calibration slope 0.84. The developed model showed acceptable calibration as shown in Figure 5. All performance measures and predictor coefficients are summarised in Table 3. The decision curve in Figure 5 shows the same net benefit between the prediction model and “treat all” approach for threshold probabilities below 10% and a higher net benefit of the developed prediction model for threshold probabilities greater than 10%.

**TABLE 3:** Developed clinical prediction models with outcomes evaluated at a 1‐month follow‐up.

Predictors	Coefficient	Model performance	Optimism‐adjusted model performance
*Responders model*			
Intercept	−2.14	Nagelkerke's *R* ^2^: 0.80	0.76
Treatment expectation (positive)	17.24	Brier score: 0.06	0.07
Pain mouth opening (≥ 2)	2.17	c‐Statistic: 0.97	0.96
CSI	−0.002		
Treatment expectation pos x CSI	−0.37		
*Pain model*			
Intercept	3.09	*R* ^2^: 0.71	0.680
Treatment expectation (positive)	110.40	RMSE: 21.70	22.90
Pain mouth opening (≥ 2)	25.10		
CSI	0.12		
Treatment expectation pos x CSI	−1.90		
*Function model*			
Intercept	4.05	*R* ^2^: 0.32	0.29
MMO	−0.07	RMSE 1.28	1.31
Pain duration*	−0.28		

*Note:* * Log‐transformed.

**FIGURE 6:**
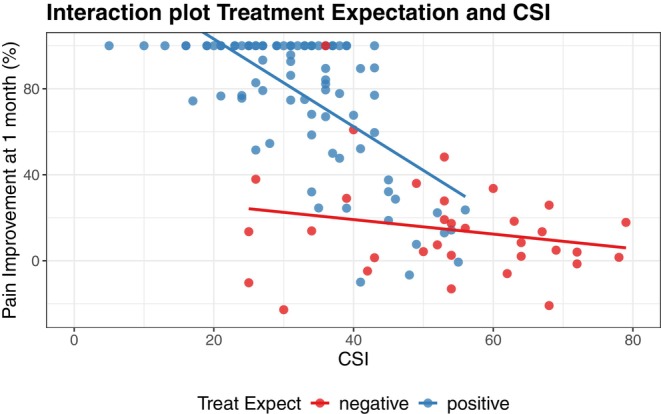
Interaction between treatment expectation and CSI in predicting pain improvement at one month follow‐up.

Percentage of pain improvement also was predicted starting from the same variable of the externally validated model and including in the final model CSI, treatment expectations, pain during MMO and the interaction term between CSI and treatment expectations. After optimism adjustment, the *R* ^2^ was 0.68, the RMSE 22.9, the intercept was 0.44 and the slope 0.99. The relationship between CSI and treatment expectations in predicting pain improvement is presented in Figure 6, showing how pain improvement depends on CSI when treatment expectations are positive, but not when treatment expectations are negative.

Finally, the prediction model for functional improvement included MMO evaluated at baseline and pain duration, included after logarithmic transformation. Performance values showed an *R* ^2^ of 0.29, a RMSE of 1.31 after optimism adjustment, an intercept of 0.03 and a slope of 0.99. A summary with estimated coefficients, original and optimism adjusted performance values for all three models is reported in Table 3.

## 4 Discussion

This study aimed to externally validate the clinical prediction models developed in our previous work, using a new cohort of patients diagnosed with TMDs [22]. An additional follow‐up was also included one month after completion of the MT intervention (one‐month follow‐up), to extend the scope of the original investigation [24]. Overall, the findings support the original prediction model in identifying patients more likely to experience a significant reduction in pain intensity immediately after the intervention. Specifically, the combination of four baseline characteristics—pain intensity greater than 2/10 during MMO, positive expectations of outcome following MT, pain localised in the craniocervical region, and lower CSI scores—was able to discriminate between patients with good and poor post‐treatment outcomes. The actual model demonstrated high discriminative ability, with a c‐statistic of 0.95, slightly higher than the optimism‐adjusted value reported in the original study (c‐statistic = 0.89). Calibration results showed acceptable agreement between predicted and observed outcomes, although with a large confidence interval. It is interesting to note that our findings support the validity of the original model, even though the two cohorts were moderately different, with the largest differences observed in age and CSI scores. The validation cohort included a slightly older population and higher CSI scores compared to the sample in the original study.

Decision curve analysis (Figure 3) confirmed that the prediction model offers superior clinical utility compared to the strategies of treating all patients or treating none. Across the entire pre‐specified range of clinically relevant thresholds (10%–40%), the clinical prediction model consistently provided a higher net benefit than both treating everyone and treating no one. This indicates that for any given threshold in this range (e.g., if a clinician decides treatment is worthwhile for a patient with at least a 25% chance of success) using the model to inform treatment decisions leads to better outcomes than treating all patients indiscriminately. This reinforces that the model is not just statistically accurate, but clinicians should use this tool alongside their current clinical reasoning to make more informed decisions [52].

The model provides additional, evidence‐based guidance to help identify patients who may respond well to MT. The predictive performance for pain intensity and functional improvement (Figure 4) also showed good overall fit, with similar values to the optimism‐adjusted overall fit and error of the original study. The pain intensity model particularly demonstrated accurate predictions, aligning closely with actual improvements observed after the MT intervention. However, the functional improvement model required minor recalibration, improving its accuracy after updating the intercept. These findings support the validity of the original model and suggest that it may be used in similar populations of patients with TMDs.

Our results showed that the key predictors identified in the original study remained relevant even one month after the end of the MT intervention (i.e., at the one‐month follow‐up) (Figure 5). This consistency at a later time point suggests that the model may be capturing core prognostic features rather than factors influencing only immediate responses with merely transient effects. Moreover, whereas previous studies investigating predictors of outcomes in musculoskeletal disorders such as neck or low back pain [20, 21], focused mainly on physical examination findings and clinical tests to define patient subgroups, our model incorporates both clinical (e.g., pain with jaw movement) and psychosocial factors (e.g., expectations, CSI). This multidimensional approach may increase the strength of our model, particularly in a population like patients with TMDs, where pain is known to be influenced by a complex interplay of somatic and psychological components [8, 9, 10]. While larger and more heterogeneous samples are still needed to strengthen external generalisability, the consistent predictive value of our model over time and across cohorts is a promising step towards developing prediction tools in the field of TMDs.

A new clinically relevant finding of our study was the highly significant interaction between treatment expectation and CSI scores when predicting pain improvement at the one‐month follow‐up (Figure 6). Patients with high positive expectations of outcome following MT experienced better outcomes when CSI scores were low, whereas CSI was irrelevant when treatment expectation was negative. New evidence suggests that CSI reflects more psychological hypervigilance rather than a central sensitisation syndrome [53]. This could explain why we observed a strong relationship with treatment expectation, often related to different psychological factors [54]. However, high CSI scores could be indicative of central sensitisation and often predict poorer responses to traditional treatment modalities in different conditions such as fibromyalgia, chronic low back pain, and chronic neck pain [55]. Our study extends this body of evidence to the field of TMDs, showing that high CSI scores may similarly moderate treatment responses in this patient population. Interestingly, the interaction showed lower improvement when CSI scores were slightly above 40, which is the typical cut‐off to indicate the presence of central sensitisation [49].

The role of treatment expectations has been investigated in various musculoskeletal disorders, and it is known to influence patient motivation, adherence, and the perceived effectiveness of interventions [56]. Positive expectations likely improve treatment responses through psychological mechanisms such as increased motivation, greater adherence to recommended treatments, and a more favourable interpretation of pain and discomfort [57]. Our findings emphasise the importance of considering patient expectations during clinical evaluations and interventions for people with TMDs, highlighting their potential as modifiable predictors that can be actively addressed through patient education, therapeutic alliance building, and shared decision‐making.

The key issue is not just the CSI scores or treatment expectations alone, but their deep interactions in predicting pain improvement. Our findings show that pain improvement depends on CSI when treatment expectations are positive, but not when treatment expectations are negative. As far as we are aware, this is the first study to explore this specific interaction and its potential predictive value for pain response in musculoskeletal disorders. A possible explanation is that positive expectations may enable patients with lower psychological distress (reflected by low CSI scores) to fully engage with treatment, amplifying its effects. In contrast, when expectations are negative, this facilitative pathway may be blocked, and even patients with low CSI scores do not experience meaningful improvement. One clinical advantage of this finding is the velocity of clinical decision‐making: patients with high treatment expectations and low CSI scores can be quickly identified as responders to MT, enabling rapid and more informed therapeutic choices. Also, this again highlights the need to foster realistic yet hopeful expectations, especially in patients with high CSI scores. Addressing expectations early could enhance the overall effectiveness of treatments for TMDs.

In recent years, musculoskeletal research has increasingly focused on identifying predictors of treatment outcomes, aiming to enhance personalised care and clinical decision‐making. For example, in their 2021 study [58], Artus et al. developed and externally validated prediction models for pain intensity outcomes in patients with low back and neck pain. Like our study, their approach combined clinical and psychological variables, reinforcing the idea that multidimensional assessment leads to better predictive accuracy. Notably, they identified pain duration, baseline pain intensity, depressive symptoms, and patient expectations as key predictors [58]. These factors closely align with those found in our cohort of people with TMDs. Concerning TMDs, Su et al. [52] developed a prediction model for guiding treatment decisions, incorporating demographic and symptom‐related variables. Their model identified several factors (i.e., anxiety, depression, sleep bruxism, pain‐ and function‐related TMD signs, stress levels, and the passive range of mouth opening) as significant predictors for determining whether a patient would benefit more from no treatment, physical therapy alone, or a combined physical and psychological approach [52]. Compared to Su et al., our model adds value by validating predictors against actual post‐treatment outcomes and including psychosocial measures such as treatment expectations and central sensitisation. Moreover, while their model aimed to guide general treatment direction, our study focused on predicting the response to a specific intervention (i.e., MT), thereby enhancing the clinical applicability and precision of decision‐making in practice. Prodoehl et al. [59] offered further insight by exploring the predictors of physical therapy visit frequency and patient satisfaction in people with TMDs. Although their outcomes differ from ours, the emphasis on psychological and behavioural factors supports our findings and the relevance of integrating patient expectations in the clinical context during treatment planning. Additional longitudinal studies have focused on identifying baseline predictors of persistent TMD pain regardless of treatment type. Forssell et al. [60] and Kapos et al. [61] found that psychological distress, somatic symptoms, and widespread pain were associated with chronicity and higher future pain intensity. These findings emphasise the prognostic importance of central mechanisms, represented in our study by the CSI, in influencing outcomes.

### 4.1 Methodological Considerations

This study has some limitations that should be noted. First, the study design does not allow us to distinguish between prognostic factors of good recovery (which predict outcomes regardless of treatment) and treatment effect modifiers (which specifically identify those patients who respond to MT). Although both are clinically relevant, this distinction is essential, and it would require randomised controlled trials in order to be fully addressed. Despite this, the model provides valuable clinical information. Knowing which patients are unlikely to improve helps clinicians avoid wasting time and resources on this specific treatment. Moreover, since MT is a low‐risk and low‐cost intervention, there is no significant downside to treating a patient who may have improved anyway due to the natural course of their condition.

The study took place in a single centre, which may limit the generalisability of the results to other clinical settings. With 124 participants, the sample size was relatively small for an external validation study. We did not meet the ≥ 100 events and ≥ 100 non‐events rule‐of‐thumb, which likely contributed to wider 95% CIs (particularly for calibration metrics) and potentially less stable estimates of the original model's performance. Despite the strong discrimination performance, a calibration slope of 1.21 suggests that the model risks resulting in an overestimation of predicted probability for patients with low probability of good outcome and underestimation of the predicted probability for patients with high probability of good outcome. Together with a large confidence interval, the limitations due to calibration suggest that the prediction model should be considered with caution. We have transparently reported this uncertainty, and these results can be used to inform the sample size calculations for future multi‐centre and larger validation studies. In addition, all assessments were conducted by an independent physiotherapist who did not deliver the MT interventions, limiting treatment‐allegiance/performance bias. However, the assessor was not blinded to baseline predictor data and also measured the primary and secondary outcomes at each time point, which may have introduced detection/observer‐expectancy bias.

Both model development and external validation were undertaken in the same dental hospital, involving the same clinicians with extensive expertise in TMDs and MT. Although this homogeneity likely contributed to the strong discrimination performance and supports the reproducibility of the prediction model, it also constrains transportability to settings that differ in care pathways, patient characteristics, referral patterns, clinician and therapist skill mix, or resource availability. Clinicians considering use of this model in other contexts should therefore apply it recognising that its predictive accuracy may differ outside the setting in which it was developed and validated. To establish transportability, future research should evaluate the model in multicentre studies across different geographic regions, populations, and clinical settings.

Despite these limitations, several methodological strengths support the reliability and potential clinical utility of our study. We externally validated previously developed clinical prediction models using a new and independent cohort of patients with TMDs, thereby increasing the robustness and clinical relevance of the findings. The inclusion of a one‐month follow‐up provided a broader perspective on treatment response and allowed for the evaluation of more sustained improvement beyond immediate effects. This is particularly relevant in the context of chronic pain, where short‐term changes may not always reflect longer‐term benefit [62]. Another strength is the multidimensional nature of the predictors used. By integrating clinical features (such as pain during jaw movement and mouth opening limitation) with psychosocial variables (such as central sensitisation and treatment expectations), our model reflects the complex biopsychosocial nature of TMDs [8, 9, 10]. Importantly, the predictors identified are easy to assess in routine clinical settings and require minimal time and resources. Finally, the model, which can be easily implemented in clinical practice using the developed nomogram [22], may be converted into a digital app to enhance usability and support clinical decision‐making.

### 4.2 Clinical Implications

The prediction models validated in this study can support clinical decision‐making in the management of people with TMDs by quickly identifying patients more likely to benefit from MT. All predictors used in the models are simple to assess and feasible for routine clinical use. A nomogram has already been developed to facilitate integration into clinical practice, providing a visual and intuitive tool for estimating the likelihood of treatment success based on individual patient characteristics [22]. To further support clinical adoption, a web‐based application has been developed to allow clinicians to enter individual patient data and instantly obtain a personalised prediction [for full details please visit https://cpr‐spine‐uob.shinyapps.io/mtfortmd/]. This tool aims to further facilitate fast and informed clinical decisions.

The interaction between treatment expectations and CSI suggests that clinicians should assess these factors together, recognising that patients with high CSI scores may have limited mid‐term improvement, even if they hold positive expectations. Furthermore, identifying a patient with a predicted poor outcome using this model can inform the clinician of a potentially more complex clinical presentation. For these patients, rather than MT alone, a multimodal approach that directly addresses the central components of their pain might be more indicated.

## 5 Conclusion

This study externally validated a clinical prediction model for patients with TMDs following a MT intervention, confirming its ability to identify individuals more likely to experience short‐term improvement in pain and function. Notably, the predictors pain during mouth opening, treatment expectations, and central sensitisation showed high accuracy in a new model at a one‐month follow‐up, suggesting their potential utility beyond immediate effects. The significant interaction between treatment expectation and CSI introduces a novel perspective on how these two factors may work together to shape clinical outcomes. Future research with a larger sample size and control group is needed to confirm the role of the identified predictors as treatment effect modifiers for MT versus general prognostic factors and improve the precision of the model performance.

## Conflicts of Interest

The authors declare no conflicts of interest.

## Supporting information


**File S1:** joor70092‐sup‐0001‐FileS1.pdf.

**File S2:** joor70092‐sup‐0002‐FileS2.docx.

**File S3:** joor70092‐sup‐0003‐FileS3.docx.

**File S4:** joor70092‐sup‐0004‐FileS4.docx.

**File S5:** joor70092‐sup‐0005‐FileS5.docx.

## Data Availability

The data that support the findings of this study are available on request from the corresponding author. The data are not publicly available due to privacy or ethical restrictions.
