# Supplementary material for: External Validation of a Clinical Prediction Tool for the Use of Manual Therapy in Patients With Temporomandibular Disorders
Source: J Oral Rehabil. 2025 Nov 11;53(2):515–28. doi: 10.1111/joor.70092 (PMC12813515; doi:10.1111/joor.70092)
Supplement: Supplementary file 2 — File S2: joor70092‐sup‐0002‐FileS2.docx. [file JOOR-53-515-s001.docx]

| **Table S2.** Comparison of demographic and predictor variables between participants included in the final analysis and those who dropped out. | | | |
| --- | --- | --- | --- |
|  | **Complete-cases (n = 124)** | **Drop-out (n = 5)** | **p-value** |
| Age | 38 (18 – 93) | 52 (26 – 67) | 0.20 |
| Gender | 94F / 30M (76% / 24%) | 3F / 2M (60% / 40%) | 0.59 |
| CSI | 36 (5 – 79) | 34 (19 – 43) | 0.57 |
| Treatment expectation | 91Pos / 33 Neg (73% / 27%) | 4 Pos / 1 Neg (80% / 20%) | 1 |
| Number pain location | 2 (1 – 6) | 2 (1 – 4) | 0.59 |
| MMO | 31 (15 – 59) | 29 (26 – 45) | 0.82 |
| Pain duration | 180 (7 – 3650) | 120 (60 – 730) | 0.87 |
| Pain mouth opening | 4 (0 – 10) | 5 (0 – 8) | 0.62 |
