# Supplementary material for: External Validation of a Clinical Prediction Tool for the Use of Manual Therapy in Patients With Temporomandibular Disorders
Source: J Oral Rehabil. 2025 Nov 11;53(2):515–28. doi: 10.1111/joor.70092 (PMC12813515; doi:10.1111/joor.70092)
Supplement: Supplementary file 3 — File S3: joor70092‐sup‐0003‐FileS3.docx. [file JOOR-53-515-s002.docx]

| **Table S3.** Univariate model performance for pain during mouth opening with different predictor codings (dichotomous, continuous, spline) | | | |
| --- | --- | --- | --- |
|  | Dicothomised | Continuous | Splines |
| Nagelkerke’s R^2^ | 0.56 | 0.41 | 0.51 |
| c-statistic | 0.84 | 0.82 | 0.85 |


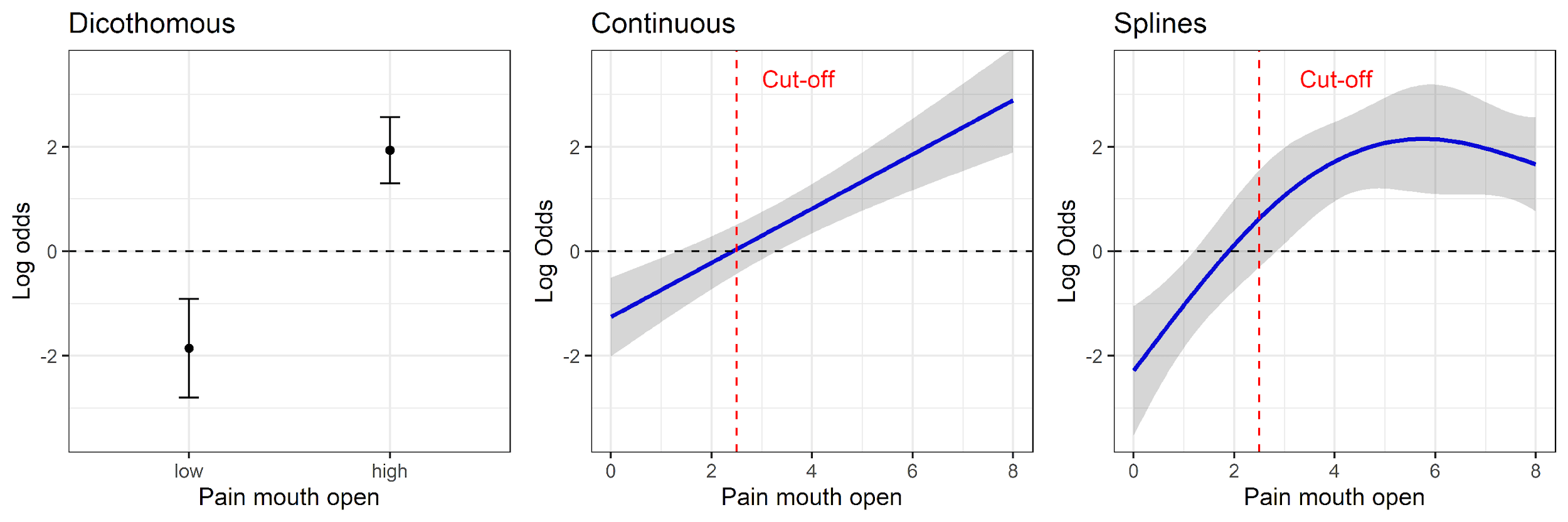


***Figure S3.*** *Univariate logistic regression of pain during mouth opening with three alternative predictor codings. Left: dichotomous variable (≤ 2 vs > 2 on a 0–10 scale) showing log-odds estimates and 95% confidence intervals. Middle: continuous 0–10 scale with fitted log-odds line (blue) and 95% confidence band; the red dashed line marks the clinical cut-off of 2. Right: restricted cubic spline fit with corresponding confidence band and the same cut-off indicated. These plots illustrate how the relationship between pain during mouth opening and the outcome is represented when the predictor is modelled as dichotomous, continuous, or using splines.*
