# Supplementary material for: External Validation of a Clinical Prediction Tool for the Use of Manual Therapy in Patients With Temporomandibular Disorders
Source: J Oral Rehabil. 2025 Nov 11;53(2):515–28. doi: 10.1111/joor.70092 (PMC12813515; doi:10.1111/joor.70092)
Supplement: Supplementary file 5 — File S5: joor70092‐sup‐0005‐FileS5.docx. [file JOOR-53-515-s003.docx]

| **Table S5.** Model performance in the primary analysis and in best- and worst-case sensitivity analyses for missing data | | | |
| --- | --- | --- | --- |
| **Metrics** | **Original model (95 % CI)** | **Worst case (95 % CI)** | **Best case (95 % CI)** |
| c-statistic | 0.95 (0.90 to 0.99) | 0.91 (0.85 to 0.97) | 0.94 (0.89 to 0.99) |
| Nagelkerke’s R^2^ | 0.75 | 0.67 | 0.73 |
| Calibration-in-the-large | -0.35 (-0.94 to 0.25) | -0.66 (-1.22 to -0.11) | -0.23 (-0.83 to 0.37) |
| Calibration slope | 1.21 (0.77 to 1.65) | 1.00 (0.65 to 1.36) | 1.08 (0.70 to 1.45) |
| Brier Score | 0.076 | 0.101 | 0.080 |


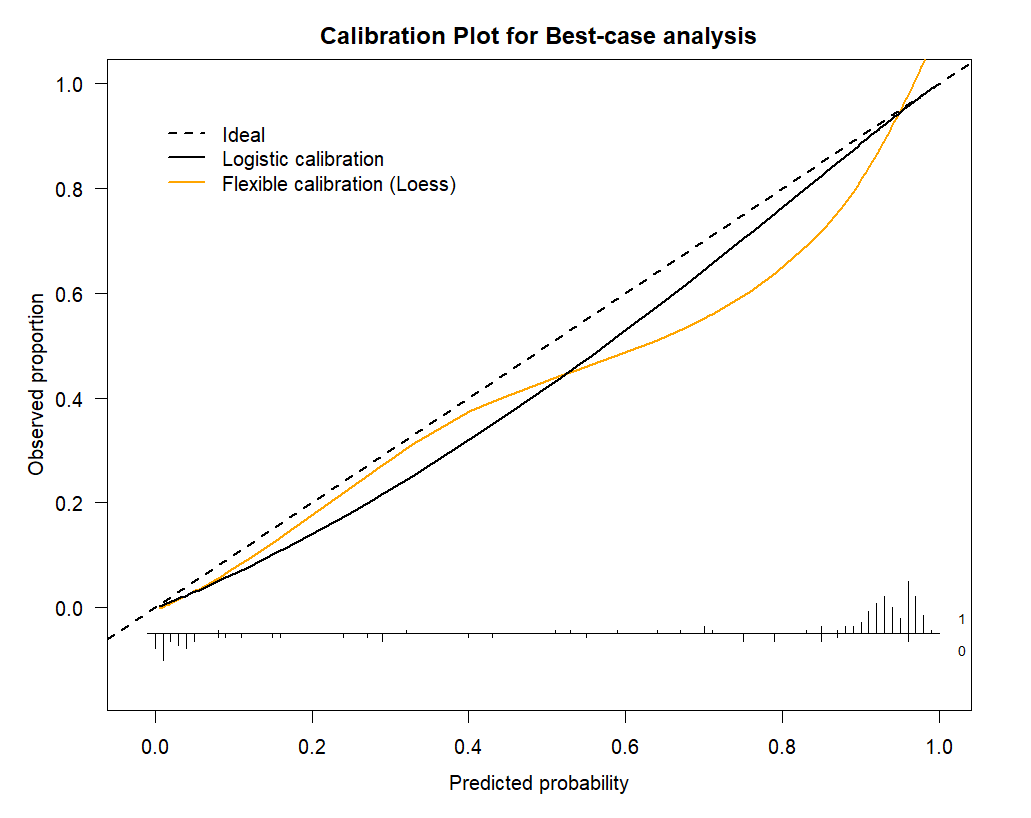

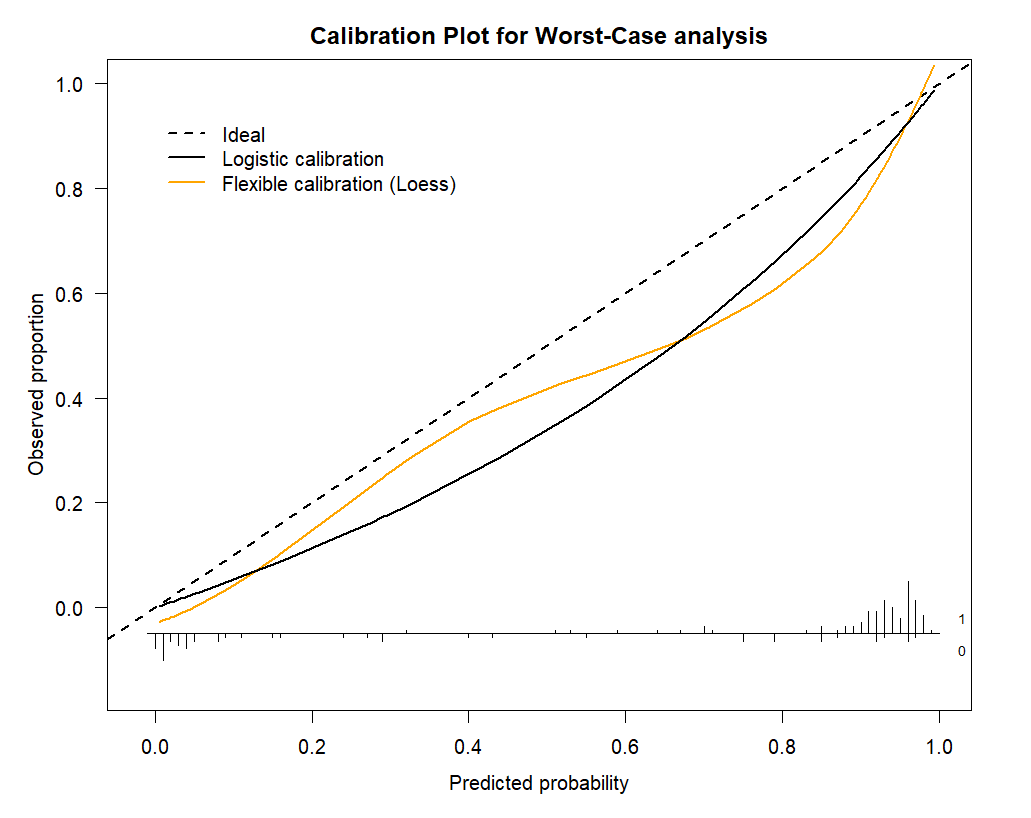


***Figure S5.*** *Calibration plots for the best- and worst-case sensitivity analyses of the prediction model. Each panel compares predicted probabilities with observed event proportions. The dashed line indicates perfect calibration (ideal), the black solid line shows logistic-regression calibration, and the orange line represents flexible loess calibration.*
